# *Drechslerelladaliensis* and *D.xiaguanensis* (Orbiliales, Orbiliaceae), two new nematode-trapping fungi from Yunnan, China

**DOI:** 10.3897/BDJ.10.e96642

**Published:** 2022-12-16

**Authors:** Fa Zhang, Saranyaphat Boonmee, Jutamart Monkai, Xiao-Yan Yang, Wen Xiao

**Affiliations:** 1 Center of Excellence in Fungal Research, Mae Fah Luang University, Chiang Rai, Thailand Center of Excellence in Fungal Research, Mae Fah Luang University Chiang Rai Thailand; 2 Institute of Eastern-Himalaya Biodiversity Research, Dali University, Dali, China Institute of Eastern-Himalaya Biodiversity Research, Dali University Dali China; 3 School of Science, Mae Fah Luang University, Chiang Rai, Thailand School of Science, Mae Fah Luang University Chiang Rai Thailand; 4 Key Laboratory of Yunnan State Education Department on Er’hai Lake Basin Protection and the Sustainable Development Research, Dali University, Dali, China Key Laboratory of Yunnan State Education Department on Er’hai Lake Basin Protection and the Sustainable Development Research, Dali University Dali China; 5 The provincial innovation team of biodiversity conservation and utility of the three parallel rivers from Dali University, Dali University, Dali, China The provincial innovation team of biodiversity conservation and utility of the three parallel rivers from Dali University, Dali University Dali China

**Keywords:** carnivorous fungi, constricting rings, new species, Orbiliaceae, taxonomy

## Abstract

**Background:**

Nematode-trapping fungi are a highly specialised group in fungi and are essential regulators of natural nematode populations. At present, more than 130 species have been discovered in Zygomycota (Zoopagaceae), Basidiomycota (*Nematoctonus*), and Ascomycota (Orbiliaceae). Amongst them, nematode-trapping fungi in Orbiliaceae have become the research focus of carnivorous fungi due to their abundant species. During the investigation of carnivorous fungi in Yunnan, China, four fungal strains isolated from burned forest soil were identified as two new nematode-trapping species in *Drechslerella* (Orbiliaceae), based on multigene phylogenetic analysis and morphological characters.

**New information:**

*Drechslerelladaliensis* sp. nov. is characterised by its ellipsoid, 1–2-septate macroconidia, clavate or bottle-shaped, 0–1-septate microconidia and unbranched, simple conidiophores. *D.xiaguanensis* sp. nov. is characterised by fusiform or spindle-shaped, 2–4-septate conidia and unbranched, simple conidiophores. Both of them produce constricting rings to capture nematodes. The phylogenetic analysis, based on combined ITS, TEF1-α and RPB2 sequences, determined their placement in *Drechslerella*. *D.daliensis* forms a basal lineage closely nested with *D.hainanensis* (YMF1.03993). *D.xiaguanensis* forms a sister lineage with *D.bembicodes* (1.01429), *D.aphrobroch*a (YMF1.00119) and *D.coelobrocha* (FWY03-25-1).

## Introduction

Nematode-trapping fungi are important predators that capture nematodes by specialised trap structures (Barron 1977, Li et al. 2006, Swe et al. 2011, Zhang and Hyde 2014). They play vital roles in maintaining energy balance and nutrient cycles in soil ecosystems and exhibit great potential for biocontrol application in agricultural management (Cooke 1962, Ulzurrun and Hsueh 2018, Zhang et al. 2020a). Most nematode-trapping fungi belong to Orbiliaceae, which have been extensively studied due to their abundant species and sophisticated trapping devices (Linford et al. 1938, Jaffee et al. 1993, Wolstrup et al. 1996, Jaffee et al. 1998, Morton et al. 2003, Liu et al. 2009, El-Borai et al. 2011, Kumar et al. 2011, Swe et al. 2011, Vilela et al. 2012). Currently, 116 predatory species in Orbiliaceae have been reported (Glockling and Dick 1994, Li et al. 2006, Wu et al. 2012, Li et al. 2013, Liu et al. 2014, Zhang and Hyde 2014, Quijada et al. 2020, Zhang et al. 2020, Zhang et al. 2020b, Zhang et al. 2022). They are classified into three genera according to their types of trapping structure: 1) *Arthrobotrys* (67 species), catching nematodes using adhesive networks; 2) *Dactylellina* (34 species), capturing nematodes by adhesive knobs, adhesive branches and non-constricting rings and 3) *Drechslelrella* (15 species), trapping nematodes with constricting rings (Scholler et al. 1999, Li et al. 2005, Yang et al. 2007, Zhang and Hyde 2014).

*Drechslerella* was established by Subramanian (1963) with the type species *D.acrochaeta* (Drechsler) Subram. It is a small genus separated from *Monacrosporium*, based on conidia producing filamentous appendages at the apex, which are lacking in *Monacrosporium*. However, filamentous appendages are usually produced when conidia germinate and are also commonly found in some species of *Arthrobotrys*. Therefore, Liu and Zhang (1994) treated *Drechslerella* as a synonym of *Monacrosporium*, based on their similar conidial morphology. Subsequently, the generic concept of nematode-trapping fungi in Orbiliaceae was revised, based on molecular phylogenetic analysis. *Drechslerella* is characterised by producing constricting rings to capture nematodes (Liou and Tzean 1997, Pfister 1997, Ahren et al. 1998, Scholler et al. 1999, Li et al. 2005). *Drechslerella* currently includes 15 accepted species, 13 of which have been reported in China (Zhang and Mo 2006, Zhang and Hyde 2014). They mainly occur in the soil or sediment of various ecosystems such as forests, mangroves, freshwater, brackish water, heavy metal polluted areas and even in tree trunks and animal faeces (Jansson and Autery 1961, Hao et al. 2005, Mo et al. 2006, Su et al. 2007, Swe et al. 2009, Zhang and Hyde 2014, She et al. 2020, Zhang et al. 2020). In soil, *Drechslerella* species are mainly distributed in the upper litter and humus layer and closely related to the density of soil nematodes (Burges and Raw 1967, Gray and Bailey 1985, Zhang and Hyde 2014). *Drechslerella* species lack nematodes mainly by the rapid expansion of the three cells that make up the constricting ring. This method of trapping nematodes mainly by mechanical force is significantly different from that of species in *Arthrobotrys* and *Dactylellina* (capture nematodes mainly with adhesive material) (Zhang and Mo 2006, Zhang and Hyde 2014). Therefore, *Drechslerella* is the most special genus amongst Orbiliaceae NTF and it is also a key group in studying the origin and evolution of carnivorous fungi.

The studies of nematode-trapping fungi have been poorly addressed in extreme habitats (Onofri and Tosi 1992, Mo et al. 2008, Swe et al. 2008). Our previous research investigated the succession of nematode-trapping fungi after fire disturbance in forests in China (She et al. 2020). Four strains were isolated and identified as two new nematode-trapping fungi in Orbiliaceae. The aim of this study is to introduce these two new species, *D.daliensis* and *D.xiaguanensis*, based on morphology and phylogenetic evidence. The discovery of these two species increased the diversity of nematode-trapping fungi and provided more valuable materials for studying the evolution and origin of carnivorous fungi, as well as more potential species for the biological control of plant and animal parasitic nematodes.

## Materials and methods

### Samples collection, isolation and morphology

The soil samples were collected from a burned forest in Cangshan Mountain, Dali City, Yunnan Province, China (100°07’44”N, 25°45’49”E). The sampling site information has been described by She et al. (2020). About 100 g of soil was collected from 10–20 cm depth using a 35 mm-diameter soil borer. The soil sample was placed into a zip lock bag and samples were brought back to the laboratory and stored at 4°C until processing.

The soil samples were sprinkled on corn meal agar (CMA) plates with sterile toothpicks. Free-living nematodes (*Panagrellusredivivus* Goodey) were added as bait to promote the germination of nematode-trapping fungi. After three weeks of incubation at 26°C, the plates were observed under a stereomicroscope to find the spores of nematode-trapping fungi. A single spore was transferred to a fresh CMA plate using a sterile toothpick, repeating this step until the pure culture was obtained.

Fungal isolates were transferred to fresh potato dextrose agar plate (PDA) using a sterile toothpick and incubated at 26°C for colony characteristics observation. The pure cultures were transferred to fresh CMA observation plates (an observation well of 2×2 cm was made by removing the agar from the centre of the CMA plate) and incubated at 26°C. When the mycelium overspread the observation well, about 500 nematodes (*P.redivivus*) were added to the well to induce the formation of trapping devices. The types of trapping devices were checked using a stereomicroscope. All morphological characters were captured and measured with an Olympus BX53 microscope (Olympus Corporation, Japan).

### DNA extraction, PCR amplification and sequencing

The genomic DNA was extracted from the mycelium grown on PDA plates according to the method described by Jeewon et al. (2002). The primer pairs ITS4-ITS5 (White et al. 1990), 526F-1567R (O'Donnell et al. 1998) and 6F-7R (Liu et al. 1999) were used to amplify the ITS, TEF1-α and RPB2 genes, respectively. The PCR amplification was performed as follows: 4 minutes of pre-denaturation at 94°C, followed by 35 cycles of 45 seconds of denaturation at 94°C, 1 minute of annealing at 52°C (ITS), 55°C (TEF1-α), 54°C (RPB2), 1.5-2 minutes of extension at 72°C and a final extension of 10 minutes at 72°C. The PCR products were purified with a DiaSpin PCR Product Purification Kit (Sangon Biotech Company, Limited, Shanghai, China). ITS and RPB2 genes were sequenced in forward and reverse directions using PCR primers and the TEF1-α region was sequenced using the 247F-609R primer pair (Yang et al. 2007) (BioSune Biotech Company, Limited, Shanghai, China).

### Phylogenetic analysis

The sequences generated in this study were compared against the NCBI GenBank database using BLASTn (BLASTn; https://blast.ncbi.nlm.nih.gov/Blast.cgi?PROGRAM=blastn&PAGE_TYPE=BlastSearch&LINK_LOC=blasthome; accessed on 16 July 2022). The morphological and BLASTn search results placed these two species in the genus *Drechslerella*. *Drechslerella* were searched in the Index Fungorum (http://www.indexfungorum.org; accessed on 16 August 2022) and Species Fungorum (http://www.speciesfungorum.org; accessed on 16 August 2022) and all relevant records were checked individually according to the relevant documents to ensure that all *Drechslerella* taxa were considered in this study (Li et al. 2013, Zhang and Hyde 2014). All reliable ITS, TEF1-α and RPB2 sequences of *Drechslerella* taxa were downloaded from the GenBank database (Table 1). The three genes datasets (including our two new species) were aligned using MAFFT online version (Madeira et al. 2022, https://www.ebi.ac.uk/Tools/msa/mafft/), then manually adjusted and linked via BioEdit v.7.2.3 (Hall 1999) and MEGA6.0 (Tamura et al. 2013). *Dactylaria* sp. YNWS02-7-1 and *Vermisporafusarina* YXJ02-13-5 were selected as outgroups (Yang et al. 2007). Phylogenetic trees were inferred with Maximum Likelihood (ML), Maximum Parsimony (MP) and Bayesian Inference analyses (BI).

SYM+I+G, GTR+I+G and GTR+I+G models were selected as best-fit optimal substitution models for ITS, TEF1-α and RPB2, respectively, via jModelTest v.2.1.10 (Posada 2008) under the Akaike Information Criterion (AIC).

MrBayes v. 3.2.6. (Huelsenbeck and Ronquist 2001) was used to perform the Bayesian Inference (BI) analysis. The multiple sequence alignment file was converted into the MrBayes compatible NEXUS file via Fasta Convert (Hall 2005). The dataset was partitioned and the optimal substitution models of each gene were equivalently replaced to conform to the setting of MrBayes. Six simultaneous Markov Chains were run for 10,000,000 generations and trees were sampled every 100 generations (a total of 100,000 trees). The first 25% of trees were discarded and the remaining trees were used to calculate the posterior probabilities (PP) in the majority rule consensus tree. All the above parameters are edited into the MrBayes block in the NEX file.

IQ-Tree v.1.6.5 (Nguyen et al. 2014) was used to perform the Maximum Likelihood (ML) analysis. The dataset was partitioned and each gene was analysed with its corresponding model. The rapid bootstrapping method with 1000 replicates (Felsenstein 1985) was used to compute the bootstrap support values (BS).

Maximum Parsimony (MP) analysis was performed via the web CIPRES Science Gateway v. 3.3 (Miller et al. 2010, https://www.phylo.org) by PAUP 4. a168 on XSEDE using the heuristic search option with 1000 random sequence additions. Max-trees were set up at 5000 and no increase. Clade stability was assessed using a bootstrap analysis with 1,000 replicates (Felsenstein 1985). Descriptive tree statistics tree length (TL), consistency index (CI), retention index (RI), rescaled consistency index (RC) and homoplasy index (HI) were calculated for all trees generated under different optimality criteria. All the above parameters are edited into the PAUP block in the NEX file.

The trees were visualised with FigTree v.1.3.1 (Rambaut 2009). The backbone tree was edited and reorganised by Microsoft PowerPoint (2013) and Adobe Photoshop CS6 software (Adobe Systems, USA). Sequences derived from this study were deposited in GenBank (Table 1).

## Taxon treatments

### Drechslerella daliensis

Fa Zhang, Xiao-Yan Yang, Kevin D Hyde sp. nov.

25550C67-0C86-508A-9865-0630E2CAEDBB

http://www.indexfungorum.org/Names:IF558120

Facesoffungi number:FOF 10565

#### Materials

- **Type status:** Holotype. **Occurrence:** occurrenceRemarks: Isolated from burned forest soil; occurrenceID: 82BE156C-BBA2-57F5-B468-EA13407B9F19; **Taxon:** scientificName: *Drechslerelladaliensis*; kingdom: Fungi; phylum: Ascomycota; class: Orbiliomycetes; order: Orbiliales; family: Orbiliaceae; genus: Drechslerella; specificEpithet: *daliensis*; taxonRank: species; scientificNameAuthorship: Fa Zhang, Xiao-Yan Yang, Kevin D. Hyde; **Location:** country: China; countryCode: CHN; stateProvince: Yunnan; county: Dali; locationRemarks: China, Yunnan Province, Dali City, Cangshan Mountain, burned forest soil, 25 July 2017; **Identification:** identifiedBy: Fa Zhang; **Record Level:** language: English; collectionID: CGMCC3.20131
- **Type status:** Isotype. **Occurrence:** occurrenceRemarks: Isolated from burned forest soil; occurrenceID: 9A5F7D25-49A6-5CC9-925B-595C9BB01673; **Taxon:** scientificName: *Drechslerelladaliensis*; kingdom: Fungi; phylum: Ascomycota; class: Orbiliomycetes; order: Orbiliales; family: Orbiliaceae; genus: Drechslerella; specificEpithet: *daliensis*; taxonRank: Species; **Location:** country: China; countryCode: CHN; stateProvince: Yunnan Province; county: Dali; locationRemarks: China, Yunnan Province, Dali City, burned forest soil; **Identification:** identifiedBy: Fa Zhang; **Record Level:** language: English; collectionID: DLU22-1

#### Description

**Figure 1.**
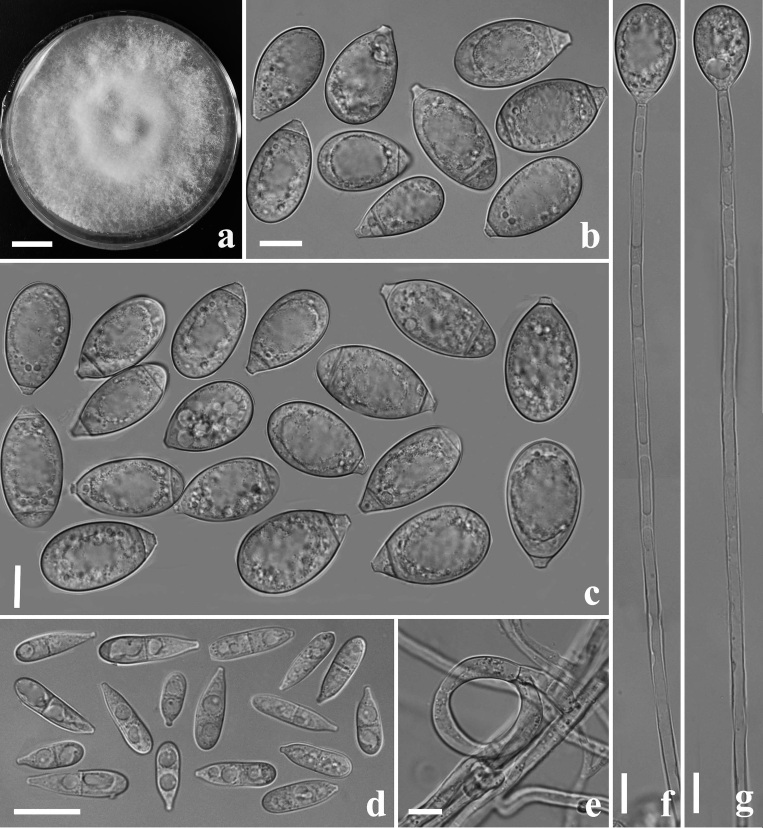
*Drechslerelladaliensis* (holotype, CGMCC3.20131). **a** Culture colony; **b, c** Macroconidia; **d** Microconidia; **e** Constricting rings; **f, g** Conidiophores. Scale bars: **a** = 1 cm; **b–g** = 10 µm.

**Colonies** white, cottony, slow-growing on PDA medium, reaching 50 mm diameter after 18 days at 26°C. **Mycelium** hyaline, septate, branched, smooth. **Conidiophores** 125–335 µm (x̅ = 216.5 µm, n = 50) long, 3–6.5 µm (x̅ = 4.5 µm, n = 50) wide at the base, 2–3.5 µm (x̅ = 3 µm, n = 50) wide at the apex, hyaline, erect, septate, unbranched, bearing a single conidium at the apex. Conidia two types: **Macroconidia** 20–49.5 × 8.5–15 µm (x̅ = 38.5–12 µm, n = 50), hyaline, smooth, ellipsoid, broadly rounded at the apex, truncate at the base, 1–2-septate, mostly 2-septate. **Microconidia** 6.5–22 × 3.5–7 µm (x̅ = 15.5–5 µm, n = 50), hyaline, smooth, clavate or bottle-shaped, broadly rounded at the apex, truncate at the base, 0–1-septate. **Chlamydospores** not observed. Capturing nematodes with three-celled **constricting rings**, in the non-constricted state, the outer diameter is 21–32 µm (x̅ = 26 µm, n = 50), the inner diameter is 12–21 µm (x̅ = 15.5 µm, n = 50), stalks 5.5–11 µm (x̅ = 8.5µm, n = 50) long and 4–6.5 µm (x̅ = 5µm, n = 50) wide (Fig. 1).

#### Diagnosis

*D.daliensis* differs from *D.hainanensis* by its thinner macroconidia and shorter microconidia.

#### Etymology

The species name “daliensis” refers to the locality (Dali) of the type specimen.

#### Distribution

China, Yunnan Province, Dali City, from burned forest soil.

### Drechslerella xiaguanensis

Fa Zhang, Xiao-Yan Yang, Kevin D. Hyde sp. nov.

5D097623-BB9F-5713-85A4-A28DD1A7E6A1

http://www.indexfungorum.org/Name:IF558121

Facesoffungi number: FOF10566

#### Materials

- **Type status:** Holotype. **Occurrence:** occurrenceRemarks: Isolated from burned forest soil; occurrenceID: 7D732B1B-4091-549C-97B3-64CC0D42FFC0; **Taxon:** scientificName: *Drechslerellaxiaguanensis*; kingdom: Fungi; phylum: Ascomycota; class: Orbiliomycetes; order: Orbiliales; family: Orbiliaceae; genus: Drechslerella; specificEpithet: *xiaguanensis*; taxonRank: Species; scientificNameAuthorship: Fa Zhang, Xiao-Yan Yang, Kevin D. Hyde; **Location:** country: China; countryCode: CHN; stateProvince: Yunnan; county: Dali; locationRemarks: China, Yunnan Province, Dali City, Cangshan Mountain, burned forest soil, 25 July 2017; **Identification:** identifiedBy: Fa Zhang; **Record Level:** language: English; collectionID: CGMCC3.20132
- **Type status:** Isotype. **Occurrence:** occurrenceRemarks: Isolated from burned forest soil; occurrenceID: A14AF229-0901-5266-92D8-8950A34DCCDF; **Taxon:** scientificName: *Drechslerellaxiaguanensis*; kingdom: Fungi; phylum: Ascomycota; class: Orbiliomycetes; order: Orbiliales; family: Orbiliaceae; genus: Drechslerella; specificEpithet: *xiaguanensis*; taxonRank: Species; **Location:** country: China; countryCode: CHN; stateProvince: Yunnan Province; county: Dali; locationRemarks: China, Yunnan Province, Dali City, Cangshan Mountain, burned forest soil; **Identification:** identifiedBy: Fa Zhang; **Record Level:** language: English; collectionID: DLU23-1

#### Description

**Figure 2.**
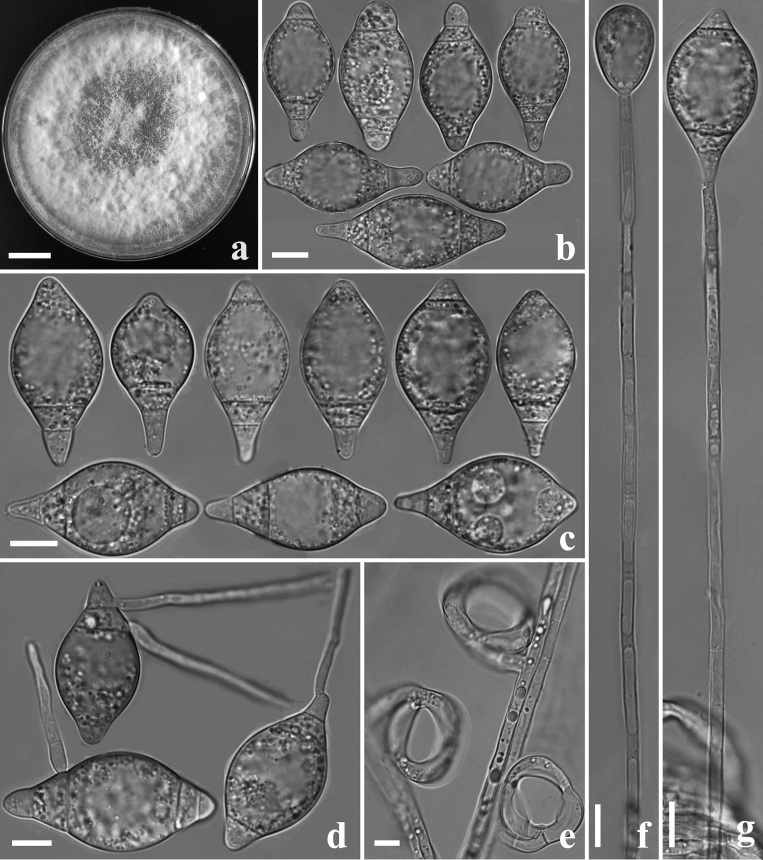
*Drechslerellaxiaguanensis* (holotype, CGMCC3.20132). **a** Culture colony; **b, c** Conidia; **d** Germinating conidia; **e** Constricting rings; **f, g** Conidiophore. Scale bars: **a** = 1 cm; **b–g** = 10 µm.

**Colonies** white, cottony, slow-growing on PDA medium, reaching 50 mm diameter after 15 days at 26°C. **Mycelium** hyaline, smooth, septate, branched. **Conidiophores** 145–315 µm (x̅ = 208.5 µm, n = 50) long, 3–6 µm (x̅ = 4 µm, n = 50) wide at the base, 2–3 µm (x̅ = 2.5 µm, n = 50) wide at the apex, hyaline, erect, septate, unbranched, bearing a single conidium at the apex. **Conidia** 33–52 × 9.5–28 µm (x̅ = 42.5–15.5 µm, n = 50), hyaline, smooth, fusiform, spindle-shaped, rounded and swollen at the both ends, 2–4-septate, mostly 3-septate, germinating tubes produced from both ends. **Chlamydospores** not observed. Capturing nematodes with three-celled **constricting rings**, in the non-constricted state, the outer diameter is 19–27.5 µm (x̅ = 24 µm, n = 50), the inner diameter is 12.5–20.5 µm (x̅ = 17 µm, n = 50), stalks 5–11.5 µm (x̅ = 9 µm, n = 50) long and 4.5–6 µm (x̅ = 5 µm, n = 50) wide (Fig. 2).

#### Diagnosis

*D.xiaguanensis* differs from *D.aphrobrocha* by its smaller conidia and swollen cells at both ends of conidia.

#### Etymology

The species name “xiaguanensis” refers to the locality (Xiaguan) of the type specimen.

#### Distribution

China, Yunnan Province, Dali City, Cangshan Mountain, from burned forest soil.

## Analysis

### Phylogenetic analyses

**Figure 3.**
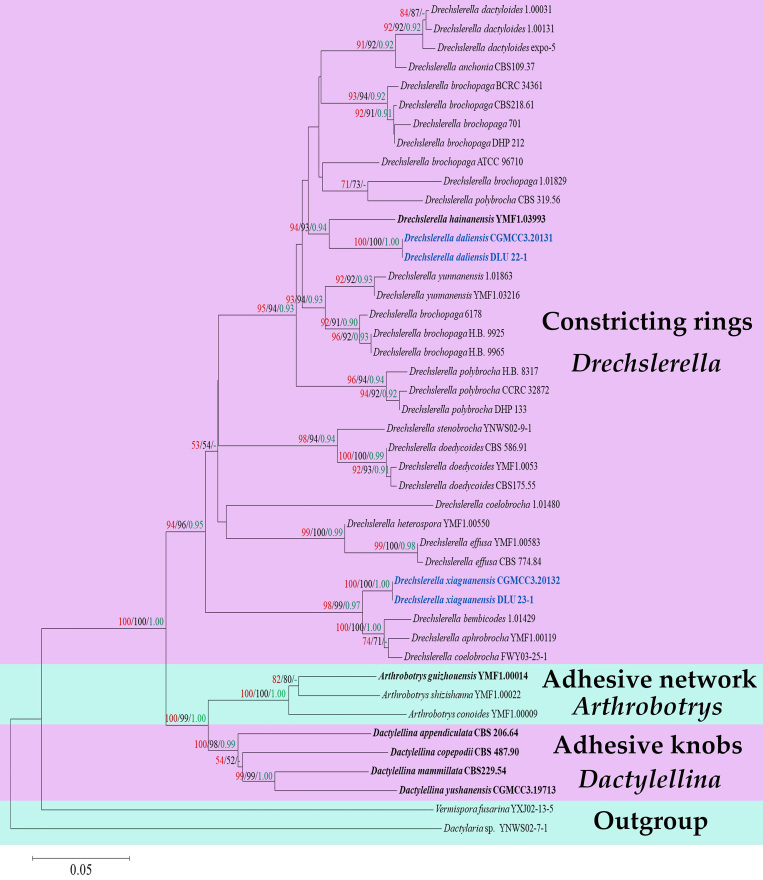
Maximum Likelihood tree, based on combined ITS, TEF1-α and RPB2 sequence data from 42 nematode-trapping taxa in Orbiliaceae. Bootstrap support values for Maximum Parsimony (red) and Maximum Likelihood (black) equal or greater than 50% and Bayesian posterior probabilities values (green) greater than 0.90 are indicated above the nodes. New isolates are in blue, ex-type strains are in bold.

A total of 15 *Drechslerella* related taxa were listed in Index Fungorum (http://www.indexfungorum.org; accessed on 16 August 2022) and Species Fungorum (http://www.speciesfungorum.org; accessed on 16 August 2022), representing 15 valid *Drechslerella* species. Amongst them, 13 species have available molecular data. The combined ITS, TEF1-α and RPB2 sequence dataset contained 42 nematode-trapping taxa in Orbiliaceae (3 *Arthrobotrys* species, 4 *Dactylellina* species and 35 *Drechslerella* taxa representing 15 species). The final dataset comprised 1939 characters (ITS = 591, TEF1-α = 534 and RPB2 = 814), including 807 conserved characters, 1072 variable characters and 748 parsimony-informative characters. After Maximum Likelihood (ML) analysis, a best-scoring likelihood tree was obtained with a final ML optimisation likelihood value of -7146.589745. For Bayesian analysis (BI), the first 25% of trees were discarded in a burn-in period, the consensus tree was calculated with the remaining trees and the Bayesian posterior probabilities were evaluated with a final average standard deviation of the split frequency of 0.009547. Within Maximum Parsimony (MP) analysis, a strict consensus tree was obtained from the two equally most parsimonious trees (TL = 2817, CI = 0.471, RI = 0.514, RC = 0.296, HI = 0.404). The trees inferred by ML, MP and BI showed similar topologies. Therefore, the best-scoring ML tree was selected for presentation (Fig. 3).

The phylogram inferred from the ITS+TEF1-α+RPB2 dataset clustered 42 Orbiliaceae nematode-trapping fungi into two large clades according to their mechanisms for catching nematodes: 1) The genus *Drechslerella* that captures nematodes by mechanical force (Zhang and Hyde 2014); 2) The genera *Arthrobotrys* and *Dactylellina* capture nematode by adhesive material (Zhang and Hyde 2014). Our two new species *D.daliensis* and *D.xiaguanensis* clustered in *Drechslerella* with high statistical support. *D.daliensis* forms a basal lineage closely nested with *D.hainanensis* (YMF1.03993) with 94% MPBS, 93% MLBS and 0.94 BYPP support. *D.xiaguanensis* forms a sister lineage with *D.bembicodes* (1.01429), *D.aphrobrocha* (YMF1.00119) and *D.coelobrocha* (FWY03-25-1) with 98% MPBS, 99% MLBS and 0.97 BYPP support (Fig. 3).

## Discussion

*Drechslerelladaliensis* and *D.xiaguanensis* produce constricting rings to capture nematodes, which is consistent with the genus *Drechslerella* (Zhang and Hyde 2014). The multi-genes phylogenetic analysis also confirmed that they are members of *Drechslerella*.

Phylogenetically, *D.daliensis* (CGMCC3.20131) forms a sister lineage to *D.hainanensis* (YMF 1.03993) with 97% MLBS, 96% MPBS and 0.95 BYPP support (Fig. 3). A comparison of ITS nucleotide shows 10.15% difference (60/591 bp) between them. Morphologically, amongst 17 species in *Drechslerella* (plus our two new species), *D.daliensis*, *D.effusa*, *D.hainanensis* and *D.heterospora* produce ellipsoid 0–3 septate conidia (Li et al. 2013, Zhang and Hyde 2014). The difference between *D.daliensis* and *D.effusa* is that the conidiophores of *D.daliensis* produce only a single conidium at the apex, while the conidiophores of *D.effusa* usually bear two or more conidia (Zhang and Hyde 2014). *D.daliensis* can be easily distinguished from *D.heterospora* by their microconidia size and the apex characteristic of conidiophore: the microconidia of *D.daliensis* are significantly smaller than those of *D.heterospora* (6.5–22 × 3.5–7 µm vs. 23–40 × 5.3–8 µm), the conidiophores of *D.heterospora* usually swollen and spherical at the apex, while those of *D.daliensis* are not swollen. In addition, *D.daliensis* does not produce chlamydospores, while *D.heterospora* produces chlamydospores in chains (Zhang and Hyde 2014). It is challenging to distinguish *D.daliensis* and *D.hainanensis* according to their shape characteristics. The difference between them is that the macroconidia of *D.daliensis* are thinner than those of *D.hainanensis* (20–49.5 × 8.5–15 µm vs. 32.5–43 × 17–25 µm) and the microconidia are shorter than those of *D.hainanensis* (6.5–22 × 3.5–7 µm vs. 18.2–22.8 × 4.2–5.3 µm) (Li et al. 2013).

In the phylogenetic analysis, *D.xiaguanensis* (CGMCC3.20131) forms a sister lineage to *D.bembicodes* (1.01429), *D.aphrobrocha* (YMF1.00119) and *D.coelobrocha* (FWY03-25-1) with 100% MLBS, 100% MPBS and 1.00 BYPP support (Fig. 3). Comparison of ITS nucleotide shows 2.6% (15/577 bp), 5.2% (30/577 bp) and 3.6% (20/556 bp) between *D.xiaguanensis* and *D.bembicodes*, *D.aphrobrocha* and *D.coelobrocha*, respectively. Morphologically, they can be distinguished by their conidia size: the conidia of *D.xiaguanensis* are thinner than those of *D.bembicodes*, shorter than those of *D.coelobrocha* and smaller than those of *D.aphrobrocha* (*D.xiaguanensis* 33–52 (42.5) × 9.5–28 (15.5) µm vs. *D.bembicodes* 36–43.2 (40) × 16.8–21.6 (20.5) µm vs. *D.coelobrocha* 45.6–55.2 (49.5) × 16.8–21.6 (19.8) µm vs. *D.aphrobrocha* 40–57.5 (51) × 15.5–35 (24.6) µm). In addition, the cells at both ends of some conidia of *D.xiaguanensis* are swollen, while *D.bembicodes*, *D.aphrobrocha* and *D.coelobrocha* are not (Drechsler 1950, Zhang and Mo 2006, Zhang and Hyde 2014). Based on the above, we propose *D.daliensis* and *D.xiaguanensis* as two new species of *Drechslerella*.

Amongst nematode-trapping fungi, species in *Arthrobotrys* are the dominant group in most ecosystems due to their strong reproductive and saprophytic ability, while the species in *Dactylellina* and *Drechslerella*, with weaker competitive abilities were rare (Jaffee et al. 1998, Hao et al. 2005, Elshafie et al. 2006, Su et al. 2007, Mo et al. 2008, Yang et al. 2008, Swe et al. 2009, Wachira et al. 2009, Yang et al. 2011). However, many species of *Dactylellina* and *Drechslerella* have been isolated from the burning forest in Cangshan, Yunnan (She et al. 2020). Amongst them, two new *Dactylellina* species (Zhang et al. 2020) and two new *Drechslerella* species (this paper) have been identified. We speculate that the reasons for this unusual phenomenon may be as follows: in normal habitat, *Arthrobotrys* species usually occupy the main living resources and are mainly distributed in the upper soil where humus, air and space are abundant due to their strong reproductive and saprophytic ability, while those species of *Dactylellina* and *Drechslerella* are mainly distributed in the lower soil where humus is scarce. When a fire occurs, *Arthrobotrys* species distributed in the upper soil are more vulnerable to the fire and are wiped out and then the habitat plaques form. In contrast, the rare species distributed in the lower layer are protected by the upper soil and preserved. In the subsequent recovery stage, these species can grow in large numbers and occupy the habitat plaque to form the dominant population in the area. Based on the above, we speculate that we would find more rare nematode-trapping fungi in burned forests. In addition, according to this principle, we speculate that other saprophytic fungi also have similar laws. Further research is underway and will be reported later.

## Supplementary Material

XML Treatment for Drechslerella daliensis

XML Treatment for Drechslerella xiaguanensis

## Figures and Tables

**Table 1.** GenBank accession numbers of isolates included in this study. The type strains are marked with T at the end of the strain number. The newly-generated sequences are indicated in bold.

Taxa	Strain numbers	GenBank accession numbers			Reference
		ITS	TEF1-α	RPB2	
*Arthrobotrysconoides*	YMF1.00009	MF948387	MF948544	MF948468	Unpublished
*Arthrobotrysguizhouensis*	YMF1.00014^T^	MF948390	MF948547	MF948471	Unpublished
*Arthrobotrysshizishanna*	YMF1.00022	MF948392	MF948549	MF948473	Unpublished
*Dactylaria* sp.	YNWS02-7-1	AY773457	AY773399	AY773428	Yang et al. (2007)
*Dactylellinaappendiculata*	CBS 206.64^T^	AF106531	DQ358227	DQ358229	Hagedorn and Scholler (1999)
*Dactylellinacopepodii*	CBS 487.90^T^	U51964	DQ999835	DQ999816	Liou and Tzean (1997)
*Dactylellinamammillata*	CBS229.54^T^	AY902794	DQ999843	DQ999817	Li et al. (2006)
*Dactylellinayushanensis*	CGMCC 3.19713^T^	MK372061	MN915113	MN915112	Zhang et al. (2020)
*Drechslerellaanchonia*	CBS109.37	AY965753	——	——	Li et al. (2006)
*Drechslerellaaphrobrocha*	YMF1.00119	MF948397	——	MF948477	Unpublished
*Drechslerellabembicodes*	1.01429	MH179731	——	MH179835	Unpublished
*Drechslerellabrochopaga*	701	AY773456	AY773398	AY773427	Yang et al. (2007)
*Drechslerellabrochopaga*	1.01829	MH179750	——	MH179852	Unpublished
*Drechslerellabrochopaga*	CBS218.61	U51950	——	——	Liou and Tzean (1997)
*Drechslerellabrochopaga*	ATCC 96710	EF445987	——	——	Smith and Jaffee (2009)
*Drechslerellabrochopaga*	DHP 212	U72609	——	——	Pfister (1997)
*Drechslerellabrochopaga*	BCRC 34361	FJ380936	——	——	Zhang et al. (2020b)
*Drechslerellabrochopaga*	H.B.9925	KT222412	——	——	Zhang et al. (2020b)
*Drechslerellabrochopaga*	H.B.9965	KT380104	——	——	Zhang et al. (2020b)
*Drechslerellabrochopaga*	6178	DQ656615	——	——	Zhang et al. (2020b)
*Drechslerellacoelobrocha*	FWY03-25-1	AY773464	AY773406	AY773435	Yang et al. (2007)
*Drechslerellacoelobrocha*	1.0148	MH179744		MH179847	Unpublished
*Drechslerelladactyloides*	1.00031	MH179690	MH179554	MH179799	Unpublished
*Drechslereladactyloides*	expo-5	AY773463	AY773405	AY773434	Yang et al. (2007)
*Drechslerelladactyloides*	1.00131	MH179705	——	MH179813	Unpublished
***Drechslerelladaliensis***	**CGMCC 3.20131**	**MT592896**	**OK556701**	**OK638157**	**This study**
***Drechslerelladaliensis***	**DLU22-1**	**OK643974**	**OK556700**	**OK638158**	**This study**
*Drechslerelladoedycoides*	YMF1.00553	MF948401	——	MF948481	Unpublished
*Drechslerelladoedycoides*	CBS 586.91	MH862283	——	——	Vu et al. (2019)
*Drechslerelladoedycoides*	CBS175.55	MH857432	——	——	Liou and Tzean (1997)
*Drechslerellaeffusa*	YMF1.00583	MF948405	MF948557	MF948484	Unpublished
*Drechslerellaeffusa*	CBS 774.84	MH861835	——	——	Vu et al. (2019)
*Drechslerellahainanensis*	YMF1.03993	KC952010	——	——	Li et al. (2013)
*Drechslerellaheterospora*	YMF1.00550	MF948400	MF948554	MF948480	Unpublished
*Drechslerellapolybrocha*	CBS 319.56	MH857657	——	——	Vu et al. (2019)
*Drechslerellapolybrocha*	CCRC 32872	U51973	——	——	Vu et al. (2019)
*Drechslerellapolybrocha*	DHP 133	U72606	——	——	Zhang et al. (2020b)
*Drechslerellapolybrocha*	H.B. 8317	KT222361	——	——	Unpablished
*Drechslerellastenobrocha*	YNWS02-9-1	AY773460	AY773402	AY773431	Yang et al. (2007)
***Drechslerellaxiaguanensis***	**CGMCC 3.20132**	**MT592900**	**OK556699**	**OK638159**	**This study**
***Drechslerellaxiaguanensis***	**DLU23-1**	**OK643975**	**OK556698**	**OK638160**	**This study**
*Drechslerellayunnanensis*	1.01863	MH179759	——	MH179861	Unpublished
*Drechslerellayunnanensi*	YMF1.03216	HQ711927	——	——	Yu et al. (2009)
*Vermisporafusarina*	YXJ02-13-5	AY773447	AY773389	AY773418	Yang et al. (2007)
